# Immunoglobulin Free Light Chains as a Biomarker of Inflammation and Heart Failure in Myocarditis and Non-Inflammatory Heart Disease

**DOI:** 10.3390/diagnostics16010050

**Published:** 2025-12-23

**Authors:** Olga Blagova, Yulia Lutokhina, Maria Kozhevnikova, Elena Zheleznykh, Evgeniya Kogan

**Affiliations:** 1 Faculty Thepapy Department No. 1, Sechenov First Moscow State Medical University (Sechenov University), 8-2, Trubetskaya Street, Moscow 119992, Russia; lutokhina_yu_a@staff.sechenov.ru; 2 Hospital Therapy Department No. 1, Sechenov First Moscow State Medical University (Sechenov University), 8-2, Trubetskaya Street, Moscow 119992, Russia; 3 Pathology Department, Sechenov First Moscow State Medical University (Sechenov University), 8-2, Trubetskaya Street, Moscow 119992, Russia

**Keywords:** immunoglobulin free light chains, myocarditis, endomyocardial biopsy, chronic heart failure, C-reactive protein, N-terminal pro-B-type natriuretic peptide

## Abstract

**Aim**: to study the level of immunoglobulin FLC in patients with myocarditis in comparison with non-inflammatory heart diseases, and FLC’s correlation with the severity of CHF. **Methods**: Ninety-nine patients (41 women, 59.6 ± 14.6 y.o.) were included in the study: 50 patients with myocarditis [confirmed by myocardial biopsy (n = 20) and/or cardiac MRI]; 49 patients with non-inflammatory heart disease. CHF was diagnosed in 66% and 65% of patients, respectively. The levels of FLC were determined using the ‘Cloneus S-FLC-K TIA Kit’ and ‘Cloneus S-FLC-L TIA Kit’ reagents. **Results**: Elevated FLC levels were found in 56% of patients with myocarditis and in 67% of comparison group patients (*p* > 0.05). Mean FLC kappa levels were 13.4 [11.7; 16.7] and 16.0 [11.3; 23.7] mg/L, FLC lambda 22.7 [16.7; 32.4] and 24.7 [18.1; 39.1] mg/L, FLC kappa/lambda ratio 0.62 [0.50; 0.73] and 0.65 [0.56; 0.76] in myocarditis and comparison groups, respectively; there were no significant differences between groups. Both groups showed correlations of FLC levels with levels of CRP and leukocytes, as well as with glomerular filtration rate, CHF NYHA class, and left ventricular ejection fraction. Only in patients with myocarditis did we observe a significant correlation between both kappa and lambda FLC and NT-proBNP (r = 0.528, *p* = 0.004, and r = 0.756, *p* < 0.001) and high-sensitivity troponin (r = 0.829, *p* = 0.042) levels. **Conclusions**: Increased FLC level may be considered an important pathogenetic link reflecting both specific mechanisms of myocarditis and severity of CHF. The determination of FLC can be used as an additional diagnostic marker, as well as one predictor of the decompensated course of myocarditis.

## 1. Introduction

Myocarditis is an inflammatory disease of the myocardium, the development of which can be caused by a variety of aetiological factors and in the implementation of which various mechanisms are involved: from the direct effect of the pathogen on cardiomyocytes, causing their death, to cardiotropic autoimmune reactions. It is not always possible to determine the aetiology of myocarditis (including infectious) and carry out aetiotropic treatment. Identifying the leading pathogenetic mechanism is therefore becoming increasingly important.

Myocardial biopsy remains the ‘gold standard’ for diagnosing myocarditis, as it allows the determination of the persistence of viruses in the myocardium and the cellular content of inflammatory infiltrates. This information is essential for choosing myocarditis treatment (antiviral, immunosuppressive therapy). However, there is still a need for non-invasive assessment of the severity of the inflammatory process. The possibilities for such assessment in clinical practice are rather limited. Common markers of inflammation [erythrocyte sedimentation rate (ESR), leukocyte levels, fibrinogen, C-reactive protein (CRP), blood ℽ-globulins] have low sensitivity in the diagnosis of subacute and chronic myocarditis [1]. The highest sensitivity (89%) and positive predictive value (81%) have been established in anticardiac antibodies [1], which are a marker of autoimmune reactions and a product of B-lymphocyte activity. The role of anticardiac antibodies has also been established in predicting a good response to immunosuppressive therapy in myocarditis [2,3].

At the same time, there is no clear correlation between anticardiac antibodies and the severity of chronic heart failure (CHF). T-lymphocytes play a leading role in the development of cellular inflammatory response in myocarditis. B-lymphocytes (CD20-positive cells) are rarely found in infiltrates; their presence indicates an unusually severe course of myocarditis and requires special treatment approaches [4]. The study of immunoglobulin free light chains (FLC) levels in the blood can provide valuable information on the activity of B cells and their involvement in the pathogenesis of humoral inflammation in myocarditis.

New guidelines for the management of myocarditis and pericarditis were presented at the ESC 2025 congress. They mention FLC among biomarkers for both diagnostic and prognostic purposes, but also state that their role requires further study [5]. Light chains (polypeptide chains consisting of 211–217 amino acid residues and having a molecular mass of approximately 22 kDa) are part of the tetrameric composition of immunoglobulins together with heavy chains and are released into the blood in small amounts (FLC). This biomarker is also of interest in terms of its possible correlations with better-studied markers of inflammation as well as with the severity of CHF [6]. Multiple studies have shown the involvement of several groups of cytokines in the pathogenesis of acute and chronic heart failure [7]. In particular, the role of C-reactive protein as a predictor of the development of CHF in patients with cardiovascular diseases has been demonstrated [8]. It has also been shown that age-related mutations in haematopoietic stem cells lead to the formation of a myeloid cell clone (clonal haematopoiesis of indeterminate potential), which is associated with systemic inflammation and adverse cardiovascular events [9]. At the same time, many drugs recommended by guidelines for the treatment of CHF have a beneficial effect on cytokine profile and inflammation [7].

Studies of FLC in myocarditis are sporadic and inconclusive [10]. However, there are data on the unfavourable prognostic value of FLC in CHF [11], which leads us to search for links between myocarditis, systemic inflammation and CHF.

The **aim** of this study was to investigate the levels of immunoglobulin free light chains in patients with myocarditis compared with non-inflammatory heart disease and their association with the severity of CHF.

## 2. Methods

The study included 99 patients (41 women and 58 men) with a mean age of 59.6 ± 14.6 years. Of these, 50 had myocarditis and 49 had non-inflammatory heart diseases.

*Laboratory tests* included a complete blood count and biochemical panel, C-reactive protein (CRP), fibrinogen, high-sensitivity cardiac troponin, N-terminal pro-B-type natriuretic peptide (NT-proBNP), anticardiac antibodies by indirect enzyme-linked immunosorbent assay, and kappa and lambda immunoglobulin FLC (Cloneus S-FLC-K TIA Kit and Cloneus S-FLC-L TIA Kit, Trimero Diagnostics, Barselona, Spain; reference values: FLC kappa 4.84–14.20 mg/L, FLC lambda 7.03–22.50 mg/L, FLC kappa/lambda ratio 0.426–1.050). NT-proBNP was measured using an electroimmunochemiluminescence method on the Cobas platform manufactured by ROCHE (Basel, Switzerland). A chemiluminescent immunoassay on microparticles (Architect technology, Abbott, Abbott Park, IL, USA) was used to assess troponin levels.

*Instrumental examination* included electrocardiography, 24-h Holter ECG monitoring and echocardiography (EchoCG, all patients), endomyocardial biopsy of the right ventricle (n = 20, haematoxylin–eosin and Van Gieson staining, immunohistochemistry for CD7, CD45, CD20, CD68-positive cells), coronary angiography (n = 56), cardiac multispiral computed tomography (n = 19) and cardiac magnetic resonance imaging (n = 37) with intravenous gadolinium enhancement.

The *diagnosis of myocarditis* was made on the basis of myocardial biopsy in 20 patients (using the Dallas criteria extended with immunohistochemical criteria; 7 patients also underwent MRI, which also showed signs of myocarditis) and in 30 patients based on cardiac MRI results (Lake Louise 2018 myocarditis criteria [8]) in combination with elevated levels of anticardiac antibodies and other clinical, laboratory and instrumental criteria. Seven patients underwent both MRI and myocardial biopsy. Endomyocardial biopsy showed lymphocytic myocarditis in all but one case (neutrophilic): active and borderline in 15 and 5 patients, respectively. The parvovirus B19 genome was detected in the myocardium of 6 patients by polymerase chain reaction and varicella zoster in one patient.

*Statistical processing* of the material was performed using IBM SPSS v.22.0. Quantitative characteristics were expressed as the mean ± one standard deviation (M ± δ) if the variable distribution was normal, or as the median with the first and third quartiles if the distribution was not normal. Normality of distribution was assessed using the Shapiro–Wilk test. The Mann–Whitney test was used to assess the statistical significance of differences in quantitative variables. Fisher’s exact test (using a 2 × 2 contingency table) was performed to compare qualitative binary variables. Differences were considered significant if *p* < 0.05. Correlations were analysed with Spearman’s coefficient. Multivariable linear regression was performed for assessment of the dependence of CHF functional class (NYHA) on the level of FLC kappa and lambda and the glomerular filtration rate (GFR, Supplementary Material S1) in a complex mathematical model. Multivariable analysis was conducted to evaluate the potential impact of GFR as a confounder that could influence FLC level.

*Ethics approval statement*. All patients signed informed consent for the study. The study was supported by the Innovative Scientific School of Sechenov University. The conduct of the study and the informed consent form were approved by the Local Ethics Committee of Sechenov University (Protocol No. 03-24 dated 8 February 2024).

## 3. Results

The *main group* consisted of 50 patients with myocarditis (20 women and 30 men, mean age 54.8 ± 13.5 years). The main manifestation of myocarditis was CHF (66% of patients): NYHA class II in 21 patients, class III in 19 patients and class IV in 3 patients; HFrEF in 20 patients; HFmrEF in 11 patients; HFpEF in 2 patients. In the remaining patients, myocarditis manifested in an isolated arrhythmic form. Constant/persistent atrial fibrillation was observed in 15 patients, paroxysmal in 4 patients. In main group, 35 patients had not received any immunosuppressive therapy at the time of enrolment. The remaining 15 patients were receiving maintenance immunosuppressive therapy with methylprednisolone 4–8 mg/day (in seven cases, in combination with azathioprine 75–150 mg/day or mycophenolate mofetil 1–2 g/day) for at least three months. The reason for including this subgroup in the study was the persistence of CHF manifestations.

The *comparison group* consisted of 49 patients with non-inflammatory heart disease (21 women and 28 men, mean age 64.6 ± 14.2 years): 13 patients suffered from coronary artery disease, 10 from acquired heart valve disease, 8 from arterial hypertension, 6 from idiopathic arrhythmias and 11 from cardiomyopathies (3—hypertrophic, 4—dilated, 4—restrictive). Patients with acute coronary syndrome or systemic infectious, immunological and oncological diseases were not included. Heart failure was diagnosed in 59% of patients: NYHA Class II in 11 patients and NYHA Class III in 18 patients; HFrEF in 10 patients, HFmrEF in 6 patients; HFpEF in 13 patients. Permanent/persistent atrial fibrillation was present in 16 patients and paroxysmal atrial fibrillation in 8 patients.

Table 1 shows the basic clinical characteristics of the patients in both groups.

Patients with myocarditis were significantly younger than patients in the comparison group, which is explained by the later development of non-inflammatory heart disease compared to myocarditis. Although the incidence of CHF was the same in both groups, the mechanisms were different. In patients with myocarditis, the main mechanism was systolic dysfunction of the left ventricle with dilatation. In the comparison group, in addition to systolic dysfunction in patients with a history of myocardial infarction, there was CHF with preserved EF (in arterial hypertension, valvular heart disease), so the mean EF in this group was higher. Mean leukocyte, ESR, CRP and fibrinogen levels remained normal in patients in both groups.

The reason for hospitalization of those patients with myocarditis who received immunosuppressive therapy was the lack of response to an optimal treatment. FLC levels were also studied in these patients as a possible marker of ongoing inflammatory activity. However, this represented only a small proportion of patients with myocarditis receiving immunosuppressive therapy. Most of them responded well to treatment and were not included in the current study. Cardiotropic therapy varied according to the nature of the underlying disease. It was most commonly used to treat heart failure and/or arrhythmias.

**Figure 1:**
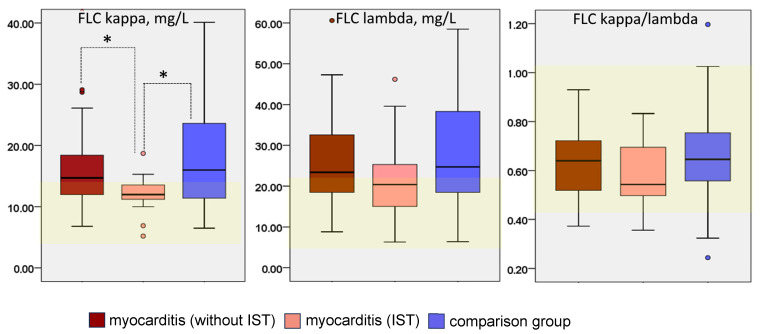
FLC level of kappa and lambda types and their ratio in patients with myocarditis without immunosuppressive therapy, with immunosuppressive therapy and in the comparison group. Burgundy columns—group of myocarditis patients without immunosuppressive therapy; pink—myocarditis patients on immunosuppressive therapy; blue—comparison group; yellow frame in each graph indicates range of normal values; *—differences between subgroups are statistically significant (*p* < 0.05).

The levels of FLC kappa and lambda and their ratio in patients with myocarditis and in the comparison group are shown in Figure 1.

In patients with myocarditis who were not receiving immunosuppressive therapy, FLC levels exceeded the normal range in 60% of patients, compared with 67% in the comparison group (differences were not significant). The lowest frequency of FLC elevation was observed in the subgroup of patients with myocarditis who were receiving immunosuppressive treatment (47%). The mean kappa and lambda FLC levels were elevated in both groups, and the kappa FLC level was significantly lower in patients with treated myocarditis than in the comparison group and in patients with untreated myocarditis. The FLC kappa/lambda ratio was within the normal range in both groups, but was lower in treated myocarditis patients (*p* > 0.05).

Further analysis was aimed at identifying factors associated with increased FLC levels. Correlation analysis showed that FLC levels of both types were closely correlated with general inflammatory markers in the blood (CRP, leukocytes, C-reactive protein, fibrinogen) as well as with CHF class and EF (more so in the myocarditis group), Table 2. A strong significant correlation with NT-proBNP and troponin levels was observed in patients with myocarditis.

**Figure 2:**
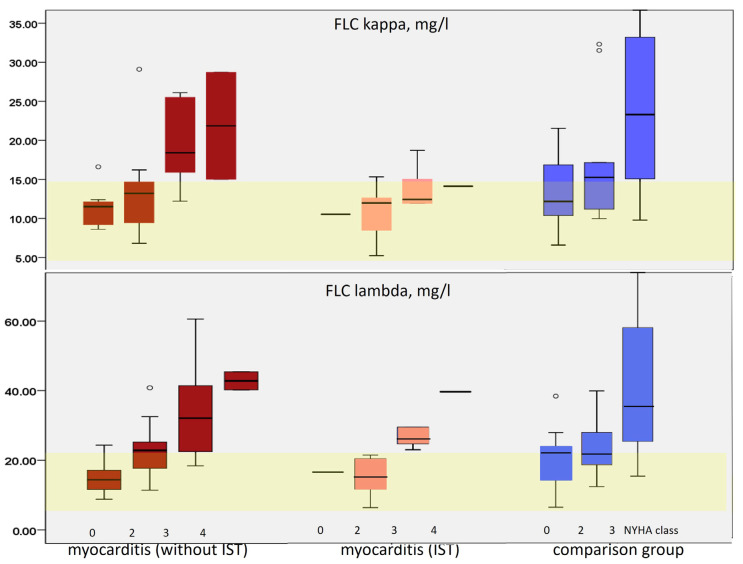
Level of kappa (**top** row) and lambda (**bottom** row) FLC depending on the severity of CHF in patients with myocarditis who did not receive immunosuppressive therapy (burgundy columns), who received it (pink columns) and in the comparison group (blue columns). Yellow frame in each graph indicates range of normal values. Horizontal lines in the myocarditis (IST) group are assuming that there were no participants with those NYHA classes in this group.

The most common relationship found in both groups was a close correlation between FLC level and CHF class, as depicted in Figure 2. In the myocarditis group, this correlation was stronger than in the comparison group (Table 2). The correlation between FLC level and CHF class was particularly clear in the subgroup of myocarditis patients not receiving immunosuppressive treatment. In myocarditis patients receiving immunosuppressive therapy, the increase in FLC level was only observed in patients with severe CHF decompensation (NYHA class III). In these patients, there was no correlation between FLC levels and the presence of CHF, which was significant only in untreated myocarditis patients (r = 0.348 for FLC kappa and 0.413 for FLC lambda, *p* < 0.05) and in the comparison group (Table 2).

## 4. Discussion

The hypothesis investigated in the present study was the possible relationship between the level of immunoglobulin FLC in the blood, a marker of B-lymphocyte activity, with the activity of myocarditis, an inflammatory heart disease. Although B lymphocytes are rarely detected in inflammatory infiltrates in myocarditis, experiments have shown that B cells are not only activated and play a pathogenic role in viral myocarditis independently of T cells, but also promote the differentiation of Th1 and Th17 cells [12]. There is also evidence that cardiomyocytes themselves can express different classes of immunoglobulins, including FLC [13].

The possible relationship between inflammation and CHF, which is not uncommon in myocarditis, was also of interest. Blood FLC levels have not been used for diagnostic or prognostic purposes in cardiology (except in cases of suspected amyloidosis). However, FLCs are one of many potential candidates for the role of a biomarker of myocardial damage. One of the first suggestions was a 2015 study [11] that included 628 patients with decompensated CHF (46% of whom had died by the end of the 3-year follow-up period). Elevated FLC levels (in 42% of subjects) were associated with a 2.38-fold increased risk of adverse outcome and remained significant in multivariate analysis, as did NT-proBNP levels, CRP and NYHA class III-IV. The authors wonder about a possible beneficial effect of CHF therapy on FLC levels (which would allow them to be used for treatment control).

The unfavourable prognostic significance of FLC levels is well known in AL-amyloidosis, where they are a direct precursor to the disease substrate [14,15]. However, the predictive value of FLC as a marker of systemic inflammation extends beyond the narrow scope of amyloidosis. Thus, it has been shown that Serum FLC is highly expressed in lung cancer and is associated with its invasion and metastasis [16]. Increased concentrations of FIC were detected in bladder cancer (in association with tumor grade) [17]. FLC are elevated in severe asthma [18]; their levels were also a strong predictor of survival in patients with chronic liver disease in the context of humoral immunity [19]. All these data indicate that FLCs can be a universal marker of immune inflammation and serve to monitor the course of immune-inflammatory diseases and other diseases with systemic inflammation.

The present study showed no significant differences between the main group (patients with myocarditis) and the comparison group (patients with non-inflammatory heart diseases) in frequency and degree of FLC elevation. At the same time, there was a significant correlation between FLC levels and the presence and severity of CHF, which was stronger in the myocarditis group. In both the main group and the comparison group, almost ⅔ of the patients had CHF. These data suggest that the increase in FLC levels is primarily associated with the presence of CHF, regardless of its aetiology and mechanisms.

The pathogenetic basis of the relationship between FLC levels and CHF is not entirely clear. In a study of patients with coronary artery disease, FLC levels were also elevated, maximally in acute heart failure and to a lesser extent in CHF [20]; such an effect was observed in patients with normal renal function and correlated with a decrease in systolic function [21]. When comparing FLC levels in myocarditis with healthy individuals, one would expect a significantly higher FLC level in patients with myocarditis, but it would be difficult to judge whether the cause of the increased FLC level is due to myocarditis proper or to CHF. This dilemma is illustrated by the only clinical study that found a significantly lower kappa and lambda FLC ratio in patients with morphologically verified myocarditis and CHF (EF less than 45%) compared to healthy controls [10]. The level of kappa-type FLC was significantly lower and lambda-type FLC significantly higher than in the group of healthy volunteers, which contradicts previous experimental data of these authors [22] and the data of C.E. Jackson et al. [11]. The authors suggest the role of a specific activation of the B-lymphocyte clone synthesising lambda-type FLCs and consider the decrease in the ratio of kappa and lambda FLCs as a marker of “myocarditis with CHF”.

Our work did not confirm monoclonal activation of FLC in patients with myocarditis or in the comparison group and is more consistent with the results of C.E. Jackson [11]. Interestingly, even in patients with AL amyloidosis, monoclonal FLC secretion had no diagnostic significance in detecting typical amyloid cardiac lesions, in contrast to BNP levels [23]. A direct toxic effect of AL chains on cardiomyocytes has been demonstrated, which is independent of the degree of amyloid infiltration of the myocardium and is undoubtedly an unfavourable prognostic factor [24,25]. By analogy with AL amyloidosis, direct damage to cytoskeletal and mitochondrial proteins under the action of FLC can be envisaged [26].

Correlations between FLC levels and troponin and NT-proBNP have been previously studied in patients with amyloidosis only [23,24]. A recent study found an interesting relationship between NT-proBNP levels and FLC in smoking patients [27]: the levels of FLC type kappa significantly decreased after smoking cessation in association with NT-proBNP due to reduction in cardiac load and a decrease in inflammation. The authors consider FLC a novel inflammatory and cardiovascular risk biomarker. A possible link in the unfavourable pathogenetic cascade in patients with cardiovascular pathology may also be kidney injury associated with an increase in FLC [28].

We obtained a strong significant correlation between FLC and NT-proBNP, which was not observed in the comparison group: this allows us to consider FLC a possible marker of a decompensated course of myocarditis. In addition, a strong correlation between FLC and troponin levels may reflect a direct role of cardiomyocyte death in the induction of the immune response (including the secondary inflammatory response). It is suggested that the involvement of FLC goes beyond general inflammatory responses and involves some specific mechanisms. Correlations with interleukin-6 levels and other markers of B lymphocyte activity should be investigated.

Among the more understandable mechanisms, our study confirmed the inverse relationship between FLC level and glomerular filtration rate, explained by a decrease in renal clearance of FLC. At the same time, the prognostic value of FLC was found to remain independent of creatinine levels [11]. Long-term persistence of FLC after acute decompensation is considered an indicator of immune dysregulation [20]. It has been shown that levosimendan administration significantly reduces FLC in CHF patients [29], which, given its mechanism of action (positive inotropic), probably reflects the influence of CHF severity on FLC rather than the reverse. In addition, patients treated with levosimendan are initially in a state of hypoperfusion and its administration improves renal function by increasing renal perfusion, which may also contribute to the rise in FLC clearance.

Finally, we found a significantly lower level of FLC in patients with myocarditis who received immunosuppressive therapy compared with untreated patients and patients in the comparison group (with no differences in CHF class). The study also included patients with myocarditis in whom the treatment did not lead to a significant reduction in heart failure, which allowed us to think about a lack of effectiveness of immunosuppressive therapy. However, the significantly lower level of FLC kappa and the lack of correlation of FLC level with the presence of CHF in this subgroup may indicate effective suppression of immune inflammation and the involvement of other (non-inflammatory) mechanisms in the maintenance of CHF. These data indicate the sensitivity of FLC to the effect of immunosuppressants and allow us to consider the level of FLC as one of the criteria of the effectiveness of myocarditis treatment.

**Figure 3:**
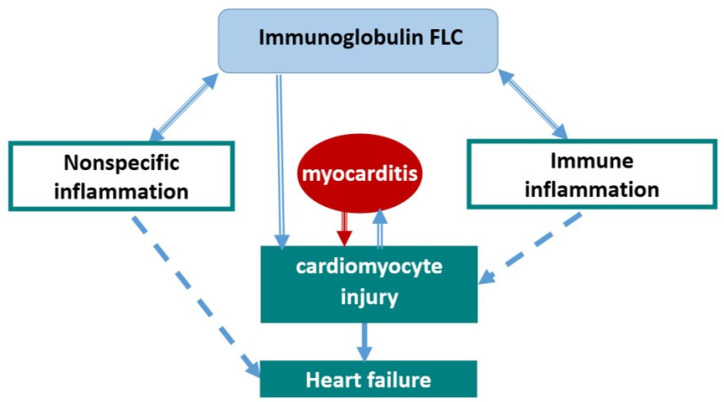
Role of immunoglobulin FLC in the pathogenesis of myocarditis and CHF.

The role of FLCs in the pathogenesis of myocarditis and CHF can be illustrated schematically as follows (Figure 3). Myocarditis appears to be a disease in which both immune (autoimmune) mechanisms and possibly a direct pathogenic effect of FLCs are realised. It can be assumed that FLCs in myocarditis are the transmitter involved in the prognostically unfavourable, decompensated variant of myocarditis and that this biomarker can be used as both a prognostic criterion and a therapeutic target. It is important to note that the mean levels of general inflammatory markers remained normal in the myocarditis group, in contrast to FLC. This means that the latter can be considered a kind of indicator (amplifier) of latent inflammation in patients with myocarditis, which makes it promising to also use them for diagnostic purposes. One of the studies showed an association between an increase in FLC and the development of atrial fibrillation—the authors conclude that this arrhythmia may have an inflammatory genesis even in the absence of CHF [10].

There have also been single experimental attempts to use FLC in the treatment of myocarditis. The group of A. Matsumori et al. in 2010 investigated the level of FLC in experimental viral myocarditis based on the relationship between mast cell activity and FLC production [30]. Not only did kappa FLC levels spontaneously increase in mice with myocarditis, but also did therapeutic administration of kappa FLC suppress the myocarditis and administration of an FLC antagonist exacerbate the disease. The issue of predicting the results of antiviral and immunosuppressive therapy for myocarditis is poorly studied, but one of the most critical for myocarditis patients. Elevated FLC levels may reflect specific mechanisms of myocarditis as well as the severity of CHF and may be evaluated as a predictor of a decompensated variant of myocarditis and an insufficient response to immunosuppressive therapy. Further investigation of this issue is required through studies with a cross-sectional design and a greater number of participants.

## 5. Study Limitations

The main limitation of the study is the relatively small number of patients enrolled. At the same time, a large number of clinical, laboratory, and instrumental parameters were entered into the database in order to assess the impact of FLC on the clinical profile of patients as comprehensively as possible. For this reason, we are cautious when interpreting the results and refrain from drawing wide-reaching conclusions. However, even with this number of patients, a series of patterns were established that allow us to define the directions of further studies: investigation of FLC levels in patients with myocarditis without significant CHF, influence of FLC levels on prognosis in patients with myocarditis and CHF, and the possibility of using them to predict and evaluate the response to therapy (both myocarditis and CHF). In future, the dynamics of FLC levels in patients with myocarditis who receive IST should be assessed using larger patient samples. Another limitation of the study is related to different GFR in subgroups, which may affect FLC levels. We performed a multivariable linear regression for assessment of the dependence of CHF functional class (NYHA) on the level of FLC kappa, lambda, and GFR (Supplementary Material S1) in a complex mathematical model. FLC lambda retained its influence on the severity of CHF among all study participants, as well as in the subgroup with myocarditis; only in the group with non-inflammatory heart diseases did the statistical significance of FLC become >0.05. One more limitation was the lack of morphological evidence for myocarditis in all patients, which prevented us from comparing FLC levels with the morphological features of myocarditis.

## 6. Conclusions

In the 2025 ESC guidelines for the diagnosis and treatment of myocarditis and pericarditis, free light chains of immunoglobulins are considered as a promising prognostic biomarker in this category of patients. However, this issue has been little studied at the moment and requires special research. In this study, the levels of immunoglobulin FLC of kappa and lambda types were elevated above the normal range in 56% of patients with myocarditis and in 67% of patients with non-inflammatory nature of CHF. In myocarditis patients receiving maintenance immunosuppressive therapy, the level of kappa-type FLC was significantly lower than in untreated patients and in the comparison group. A direct positive correlation was found between the levels of both types of FLC and the severity of CHF (NYHA functional class), both in the presence and absence of left ventricular systolic dysfunction. Correlations were also found between FLC levels and general inflammatory markers (CRP, leukocytes, ESR) and troponin levels. Only in patients with myocarditis was there a strong correlation between FLC levels and NT-proBNP. In the absence of a significant increase in the level of general inflammatory markers in the blood of patients with myocarditis, the determination of FLC can be used as an additional diagnostic marker. Its diagnostic and prognostic value in patients without CHF requires further study.

## Figures and Tables

**Table 1:** Basic clinical characteristics of patients at the time of enrolment.

Parameter	All Patients with Myocarditis	Myocarditis (Without IST)	Myocarditis (Receiving IST)	Comparison Group
n	50	35	15	49
Age, years	54.8 ± 13.5 *	56.9 ± 13.9	49.0 ± 11.5	64.6 ± 14.2 *
NYHA class	2 [1; 3]	2 [1; 3]	2 [2; 3]	2 [0; 3]
CRP, mg/mL	2.9 [1.0; 6.0]	3.1 [0.7; 5.3]	2,4 [1.1; 13.5]	2.2 [0.9; 6.5]
ESR mm/h	9 [5; 17] *	11 [5; 19]	8 [5; 11] **+**	12 [8; 21] * **+**
leukocytes, ×10^9^	6.8 ± 1.8	6.6 ± 1.6	7.3 ± 2.3	6.7 ± 1.5
neutrophils, ×10^9^	3.3 [0.5; 4.5]	3.2 [2.6; 4.5]	4.3 [2.3; 4.7]	3.5 [2.9; 4.5]
lymphocytes, ×10^9^	2.7 [1.7; 2.8]	2.3 [1.8; 2.8]	2.3 [1.8; 3.0]	2.2 [1.7; 2.5]
fibrinogen, g/L	2.9 ± 0.7	3.0 ± 0.7	2.7 ± 0.5	2.9 ± 0.7
NT-proBNP, pg/mL	992 [338; 2626]	1251 [372; 3633]	992 [180; 2355]	1315 [833; 4022]
troponin, ng/mL	14.0 [1.2; 26.8]	14.0 [1.2; 26.8]	-	-
GFR, mL/min/1.73 m^2^	67.8 ± 18.8 *	65.5 ± 19.4 ●	73.1 ± 16.5 ●**+**	58.9 ± 14.7 * **+**
FLC elevation	28 (56%)	21 (60%)	7 (47%)	33 (67%)
FLC kappa, mg/L	13.4 [11.7; 16.7]	14.7 [11.8; 19.2] ●	12.1 [9.6; 13.5] ●**+**	16.0 [11.3; 23.7] **+**
FLC lambda, mg/L	22.7 [16.7; 32.4]	23.4 [18.4; 32.6]	20.9 [14.9; 25.8]	24.7 [18.1; 39.1]
FLC kappa/lambda	0.62 [0.50; 0.73]	0.64 [0.51; 0.73]	0.53 [0.50; 0.60]	0.65 [0.56; 0.76]
AF, n (%)	22 (44%)	17 (49%)	5 (33%)	26 (53%)
low QRS voltage	12 (24%)	5 (14%)	2 (13%)	5 (10%)
LBBB	7 (14%)	5 (14%)	2 (13%)	5 (10%)
PVC/24 h	240 [10; 2000]	42 [10; 1454]	791 [222; 2117] **+**	45 [5; 260] **+**
Nonsustained VT	19 (38%)	10 (29%) ●	9 (60%) ●**+**	16 (33%) **+**
Sustained VT	4 (8%)	3 (9%)	1 (7%)	1 (2%)
IVS, cm	1.0 [0.9; 1.1]	1.1 [0.9; 1.1]	0.9 [0.8; 1.0]	1.0 [0.9; 1.2]
LV EDD, cm	5.8 ± 0.8 *	5.6 ± 0.7 ●◆	6.2 ± 0.9 ●**+**	5.2 ± 1.0 * ◆**+**
LV EDV, mL	133 [111; 183] *	128 [101; 173] ◆	145 [126; 185] +	85 [68; 130] * ◆**+**
LV ESV, mL	76 [56; 111] *	67 [52; 114] ◆	87 [66; 112] +	38 [25; 73] * ◆**+**
LV EF, %	42.9 ± 12.9 *	43.1 ± 14.0 ◆	42.3 ± 10.2 +	52.3 ± 15.1 * ◆**+**
VTI, cm	12.7 [10.1; 17.0]	12.9 [10.2; 16.9]	12.4 [10.0; 18.0]	16.8 [10.9; 18.0]
E/A	1.1 [0.8; 1.4]	1,2 [0.8; 1.4]	0,9 [0.6; 1.4]	1.2 [0.7; 2.1]
LA, cm	4.4 ± 1.1	4.2 ± 0.9 *	4.9 ± 1.3	4.3 ± 0.7
LA, mL	73 [57; 114]	74 [56; 114]	70 [61; 114]	79 [45; 92]
RA, mL	46 [34; 85]	48 [35; 77]	38 [30; 94]	52 [35; 77]
RV, cm	3.2 ± 0.6	3.2 ± 0.6	3.4 ± 0.5	3.1 ± 0.6
sPAP, mmHg	30 [26; 37]	30 [25; 36]	30 [27; 39]	30 [25; 45]
MR, grade	1 [1; 2]	1 [0.9; 2.0]	2 [1; 2]	1.0 [0.5; 2.0]
TR, grade	1 [0.5; 1.25]	1 [0.5; 1.5]	1 [1; 1]	1.0 [0.5; 2.0]
ACEi/ARBs	5/6 (22%)	3/6 (31%)	2/0 (7%)	7/11 (37%)
ARNI	27 (54%) *	17 (49%)	10 (67%) **+**	10 (20%) * **+**
β-blockers	43 (86%)	30 (86%)	13 (87%)	36 (74%)
dapagliflozin	32 (64%) *	19 (54%) ●	13 (87%) ●**+**	16 (33%) * **+**
MRA	41 (82%) *	28 (80%)	13 (87%)	30 (61%) *
loop diuretics	34 (68%)	21 (57%)	13 (87%)	27 (55%)
amiodarone	18 (36%)	11 (31%)	7 (47%)	15 (31%)

Comparison of structural and functional parameters was carried out between the following groups of patients (*p* < 0.05): *—patients with myocarditis and the comparison group; ●—patients with myocarditis who received IST and without IST; ◆—patients with myocarditis without IST and the comparison group; **+**—patients with myocarditis with IST and the comparison group. ACEi/ARBs—angiotensin-converting enzyme inhibitor or angiotensin II receptor blocker; AF—atrial fibrillation; ARNI—angiotensin receptor and neprilysin inhibitor; CRP—C-reactive protein; EDD—end-diastolic diameter (EchoCG); EDV—end-diastolic volume (EchoCG); EF—ejection fraction (EchoCG); ESR—erythrocyte sedimentation rate; ESV—end-systolic volume (EchoCG); GFR—glomerular filtration rate (by CKD-EPI); IST—immunosuppressive therapy; IVS—interventricular septum (EchoCG); LA—left atrium (EchoCG); LBBB—left bundle branch block; LV—left ventricle; MRA—mineralocorticoid receptor antagonist; MR—mitral regurgitation (EchoCG); PVC—premature ventricular contractions; RA—right atrium (EchoCG); RV—right ventricle (EchoCG); sPAP—pulmonary artery systolic pressure (EchoCG); TR—tricuspidal regurgitation (EchoCG); VT—ventricular tachycardia; VTI—velocity time integral (EchoCG).

**Table 2:** Significant correlations of FLC levels, general inflammatory markers in blood, and severity of CHF.

Parameters/Correlation Coefficient	Myocarditis (n = 50)		Comparison Group (n = 49)		All Patients (n = 99)	
	Kappa	Lambda	Kappa	Lambda	Kappa	Lambda
FLC—age	0.632 *p* < 0.001	0.647 *p* < 0.001	-	-	0.379 *p* < 0.001	0.403 *p* < 0.001
FLC—CRP	0.497 *p* < 0.001	0.496 *p* < 0.001	0.561 *p* < 0.001	0.653 *p* < 0.001	0.508 *p* < 0.001	0.565 *p* = 0.001
FLC—troponin	-	-	-	-	-	0.829 *p* = 0.042
FLC—leukocytes	0.310 *p* = 0.028	0.367 *p* = 0.009	0.305 *p* = 0.035	0.352 *p* = 0.014	0.299 *p* = 0.003	0.344 *p* = 0.001
FLC—ESR	0.362 *p* = 0.010	-	0.501 *p* < 0.001	0.584 *p* < 0.001	0.438 *p* < 0.001	0.405 *p* < 0.001
FLC—GFR	−0.550 *p* < 0.001	−0.637 *p* < 0.001	−0.327 *p* = 0.022	−0.511 *p* < 0.001	−0.473 *p* < 0.001	−0.605 *p* < 0.001
FLC—NT-proBNP	0.528 *p* = 0.004	0.756 *p* < 0.001	-	-	0.436 *p* = 0.002	0.578 *p* < 0.001
FLC—CHF presence	-	0.363 *p* = 0.010	0.479 *p* = 0.001	0.443 *p* = 0.001	0.343 *p* = 0.001	0.393 *p* < 0.001
FLC—NYHA class	0.588 *p* < 0.001	0.696 *p* < 0.001	0.528 *p* < 0.001	0.594 *p* < 0.001	0.501 *p* < 0.001	0.601 *p* < 0.001
FLC—LV EF	−0.409 *p* = 0.003	−0.501 *p* < 0.001	−0.350 *p* = 0.016	−0.312 *p* = 0.033	−0.274 *p* = 0.007	−0.306 *p* = 0.002

GFR—glomerular filtration rate (by CKD-EPI); ESR—erythrocyte sedimentation rate; CRP—C-reactive protein; LV EF—left ventricle ejection fraction (EchoCG).

## Data Availability

The original contributions presented in this study are included in the article/Supplementary Material. Further inquiries can be directed to the corresponding author.

## Supplementary Materials

The following supporting information can be downloaded at: https://www.mdpi.com/article/10.3390/diagnostics16010050/s1, The Supplementary Materials provide an assessment of the dependence of CHF functional class (NYHA) on the level of FLC kappa, lambda, and GFR using the multivariable linear regression method.

## Abbreviations

FLC, free light chains; ESR, erythrocyte sedimentation rate; CRP, C-reactive protein; EF, ejection fraction; CHF, chronic heart failure; HF, heart failure; HFmrEF, heart failure with mildly reduced ejection fraction; HFpEF, heart failure with preserved ejection fraction; HFrEF, heart failure with reduced ejection fraction; NT-proBNP, N-terminal pro-B-type natriuretic peptide.
