# Supplementary material for: Immunoglobulin Free Light Chains as a Biomarker of Inflammation and Heart Failure in Myocarditis and Non-Inflammatory Heart Disease
Source: Diagnostics (Basel). 2025 Dec 23;16(1):50. doi: 10.3390/diagnostics16010050 (PMC12785386; doi:10.3390/diagnostics16010050)
Supplement: Supplementary file 1 [file diagnostics-16-00050-s001.zip › diagnostics-3988666-supplementary.pdf]

Supplementary materials S1

Multivariable linear regression

Assessment of the dependence of CHF functional class (NYHA)

on the level of FLC kappa, lambda and GFR

1. All patients included in the study

Model p-value (ANOVA) < 0.001
R = 0.555
R-square = 0.308

**Parameter**	**B**	**Standard error**	**beta**	***T***	**p-value**	**95% CI for B coefficient**	
						**Lower bound**	**Upper bound**
Const	2.009	.642		3.127	.002	.734	3.284
FLC kappa	.004	.017	.036	.257	.798	-.030	.038
**FLC lambda**	.027	.011	.364	2.418	**.018**	.005	.050
**GFR**	-.016	.007	-.227	-2.180	**.032**	-.031	-.001

2. Patients with myocarditis

Model p-value (ANOVA) < 0.001
R = 0.644
R-square = 0.415

**Parameter**	**B**	**Standard error**	**beta**	***T***	**p-value**	**95% CI for B coefficient**	
						**Lower bound**	**Upper bound**
Const	1.570	.917		1.711	.094	-.277	3.416
FLC kappa	-.028	.028	-.188	-.978	.333	-.085	.029
**FLC lambda**	.064	.021	.657	3.060	**.004**	.022	.105
GFR	-.011	.009	-.176	-1.166	.250	-.029	.008

3. Patients with non-inflammatory heart diseases (comparison group)

Model p-value (ANOVA) < 0.001
R = 0.641
R-square = 0.411

**Parameter**	**B**	**Standard error**	**beta**	***T***	**p-value**	**95% CI for B coefficient**	
						**Lower bound**	**Upper bound**
Const	2.604	.926		2.811	.007	.738	4.469
FLC kappa	.023	.020	.209	1.155	.254	-.017	.064
FLC lambda	.014	.013	.208	1.058	.295	-.013	.041
**GFR**	-.032	.012	-.349	-2.574	**.013**	-.056	-.007

Assessment of the dependence of CHF functional class (NYHA)

on the level of FLC kappa, lambda and ESR and CRP

1. **All patients included in the study**

Model p-value (ANOVA) < 0.001
R = 0.571
R-square = 0.326

**Parameter**	**B**	**Standard error**	**beta**	***T***	**p-value**	**95% CI for B coefficient**	
						**Lower bound**	**Upper bound**
Const	.735	.218		3.367	.001	.302	1.169
FLC kappa	.008	.017	.063	.442	.659	-.027	.042
**FLC lambda**	.033	.011	.438	2.986	**.004**	.011	.055
**CRP**	.031	.012	.230	2.490	**.015**	.006	.056
ESR	-.009	.009	-.102	-.991	.324	-.026	.009

Model p-value (ANOVA) < 0.001
R = 0.523
R-square = 0.274

**Parameter**	**B**	**Standard error**	**beta**	***T***	**p-value**	**95% CI for B coefficient**	
						**Lower bound**	**Upper bound**
Const	.694	.225		3.086	.003	.248	1.140
FLC kappa	.006	.017	.045	.316	.752	-.029	.040
**FLC lambda**	.037	.011	.487	3.419	**.001**	.015	.058
